# Improvement of anemia in five dogs with nonregenerative anemia treated with allogeneic adipose-derived stem cells

**DOI:** 10.1016/j.vas.2022.100264

**Published:** 2022-07-19

**Authors:** Takuya Mizuno, Misuzu Inoue, Takeaki Kubo, Yoshihide Iwaki, Kosuke Kawamoto, Kazuhito Itamoto, Satoshi Kambayashi, Masaya Igase, Kenji Baba, Masaru Okuda

**Affiliations:** a Laboratory of Molecular Diagnostics and Therapeutics, Joint Faculty of Veterinary Medicine, Yamaguchi University, Yamaguchi, Japan; b Research & Development Center for Cell Therapy, Foundation for Biomedical Research and Innovation at Kobe, Kobe, Hyogo, Japan; c Life Sciences Business Division, FUJIFILM Corporation, Kanagawa, Japan; d Research and Development Section, Anicom Specialty Medical Institute Inc., Kanagawa, Japan; e Laboratory of Veterinary Small Animal Clinical Science, Joint Faculty of Veterinary Medicine, Yamaguchi University, Yamaguchi, Japan; f Laboratory of Veterinary Internal Medicine, Joint Faculty of Veterinary Medicine, Yamaguchi University, Yamaguchi, Japan

**Keywords:** Allogenic, Adipose-derived stem cells, Canine, Nonregenerative anemia, Mesenchymal stem cells

## Abstract

**Background:**

Nonregenerative anemia is occasionally seen in dogs and can be caused by many factors, among which nonregenerative immune-mediated anemia (NRIMA) and pure red cell anemia are relatively common causes. These are thought to be caused by immune-mediated destruction of the erythroid lineage and are treated with immunosuppressive drugs, but some of them are refractory or recurrent, so new treatments are needed.

**Objectives:**

To examine the efficacy of allogeneic adipose-derived stem cells (ADSCs) for the treatment of nonregerative anemia in dogs.

**Methods:**

ADSCs were administered to total five nonregenerative anemia cases; two NRIMA cases and two suspected NRIMA cases that were refractory to immunosuppressive agents, and one NRIMA case that has not been treated with immunosuppressive agents.

**Results:**

In all cases, anemia was improved, and blood transfusion was no longer necessary.

**Conclusions:**

This study suggests that allogeneic ADSCs may be one of the rescue therapies for the refractory immune-mediated anemia in dogs.

## Highlight

• Five canine cases with nonregenerative anemia were included in this study.
• All were treated with allogeneic adipose-derived stem cells (ADSCs).
• All cases showed improvement of anemia by ADSCs treatment.

## Introduction

Nonregenerative anemia is a relatively common condition in dogs, with known causes including inflammatory diseases, chronic renal disease, nutritional anemia, or bone marrow diseases. Among them, pure red cell anemia (PRCA) and nonregenerative immune-mediated anemia (NRIMA) are the most common bone marrow diseases (Weiss, 2006), especially in Japan where the incidence of NRIMA in miniature dachshunds is very high (Tani, Tomiyasu, Ohmi, Ohno & Tsujimoto, 2020). PRCA is thought to be caused by a lack of erythroblast progenitor cells, whereas NRIMA is thought to be caused by phagocytosis and abnormal differentiation of erythroblasts in the bone marrow (Weiss, 2008; Woolhead, Szladovits, Chan, Swann & Glanemann, 2021). Although the pathogenesis of both PRCA and NRIMA is not completely understood, treatment usually entails immunosuppressive therapy due to the possibility of immune-mediated destruction. However, the response rates and times to response vary, some cases do not respond (Woolhead et al., 2021), and no established treatment exists.

Mesenchymal stem cells (MSCs) are one type of stem cell that can differentiate and proliferate in a variety of ways, making them be one of the cell therapies in regenerative medicine. MSCs are commonly derived from bone marrow or fat tissues and have been studied in veterinary medicine (Voga, Adamic, Vengust & Majdic, 2020). They were previously thought to be a type of regenerative medicine, but in recent years, the possibility that they demonstrate immunomodulatory effects in dogs (Cristóbal et al., 2021; Kaur et al.2021), as well as humans, have been raised, and their use for immunomodulation is being researched. In Japan, in 2015, allogeneic BM-derived stem cells were approved for use in the suppression of graft *versus* host disease (GVHD) after hematopoietic stem cell transplantation in humans. In dogs, canine adipose-derived stem cells (ADSCs) have been used to treat immune-mediated diseases, such as canine atopic dermatitis (Kaur et al., 2021), inflammatory bowel disease (IBD) (Cristóbal et al., 2021), and Keratoconjunctivitis sicca (KCS) (Hermida-Prieto, García-Castro & Mariñas-Pardo, 2021) in addition to the regenerative purpose (Olsson et al., 2021; Prządka et al., 2021). Furthermore, canine allogeneic ADSCs were approved by the Ministry of Agriculture, Forestry, and Fisheries for improvement of clinical signs associated with thoracolumbar disk herniation in dogs in 2021. However, there are still few numbers of reports on the use of ADSCs for immune-mediated diseases to achieve immunosuppressive effects in dogs as well as humans.

In this study, we presented five cases of dogs with refractory or recurrent nonregenerative anemia who improved after receiving allogeneic ADSCs generated by our own.

## Materials and methods

### Preparation and stocks of allonegeic ADSCs

The adipose tissue was donated by a healthy male beagle dog, which was 2-year-old, had recommended vaccinations in Japan, and determined to be a suitable donor by veterinarians. By surgical excision, adipose tissue was collected aseptically from the abdominal region. Adipose tissue was digested with collagenase (FUJIFILM Wako Pure Chemical Corporation. Tokyo, Japan) at 50 U/0.1 g · fat/mL over 1.5 h at 37 °C. Following digestion, a sample was centrifuged at 750 × g for 10 min. The precipitate was collected and cultured in MSCGM™ Mesenchymal Stem Cell Basal Medium (Lonza K.K., Tokyo, Japan) with a density of 1000 cells/cm^2^ in a humidified incubator, at 37 °C in the presence of 5% CO_2_. After 72 h, cultures were washed with phosphate buffer saline (FUJIFILM Wako Pure Chemical Corporation) to remove non-adherent cells, and a fresh medium was added. After 7 days of culture, the cell were harvested, followed by washing with D-PBS (-), resuspended in COS banker (Cosmobio co., ltd, Tokyo, Japan), and stored as passage one stocks at −150 °C for longterm storage until clinical use. Some of the stocks were defrosted for analyzing characteristics to verify the suitability for clinical use.

Surface antigen expression on ADSCs were confirmed by flow cytometry. In brief, after thawed, ADSCs were washed with Flow Cytometry Staining buffer (Thermo Fisher Scientific Japan, Tokyo, Japan), and stained with either of antibodies; FITC-labelled anti-CD29 (TS2/16), FITC-labelled anti-CD44 (IM7), PE-labelled anti-CD90 (YKIX337.217), FITC-labelled anti-CD45 (YKIX716.13), or FITC-labelled anti-canine MHCII (YKIX334.2) antibodies (all were from Thermo Fisher Scientific Japan, Tokyo, Japan), followed by analyze usisng CyFlow space (Sysmex co., ltd., Kobe, Japan). Crossreactivity of all antibodies to canine cells were confirmed in the manufactures’ reports.

To confirm the multipotency of ADSCs, ADSCs were cultured in the mesenchymal stem cell osteogenic differentiation medium (Takara Bio Inc., Kusatsu, Japan), mesenchymal stem cell adipogenic differentiation medium 2 (Takara Bio Inc.), or Mesenchymal Stem cell Chondrogenic differentiation medium (Takara Bio Inc.) according to manufacturers’ instructions. After indicataed days of culture for differenciation, the differenciation to osteoblasts, chondroblasts, and adipocytes were confirmed with appropriate staning.

### Patient selection

Dogs were included in this clinical trial (Table 1), if (1) the dogs diagnosed as a NRIMA or the dogs who NRIMA was highly suspected by exclusion of other diseases, and (2) an anemia was refractory to conventional treatment, or the owner was required to want to participate in this trial. A diagnosis of NRIMA was based on previous study (Woolhead et al., 2021), and the test results required for diagnosis is shown in Table 1. Before being included in the study, all owners were given an oral and written explanation of the study, and they gave written consent to participate. The study was approved by the ethical committee of Yamaguchi University Animal Medical Center (approved number 5).

**Table 1:** Case summary of 5 cases.

Case No.	Breed	Age (years)	Sex	Coomb's test	Corrected reticulocyte percentage (CR%)	Spherocytosis	Total Bilirubin (mg/dl)	BM examinations	Diagnosis	Prior treatment	Concurrent immunosuppressive therapy	No. of ADSC injections
1	Miniature dachshund	12	Female	Positive	< 1.0	None	0.2	slight myelofibrosis and erythroid hypoplasia	NRIMA (refractory)	Predonisoone, MMF, Blood transfusion	Predonisoone, MMF	1
2	Miniature dachshund	9	Castrated male	Positive	< 1.0	None	0.7	normal myelopoiesis and erythropoiesis	NRIMA (refractory)	Predonisoone, MMF, Blood transfusion	Predonisoone, MMF	4
3	Miniature Pinscher	9	Female	Positive	< 1.0	None	0.1	erythroid hyperplasia with left shift and erythrophagocytosis and myeloid hyperplasia	NRIMA	None	None	1
4	Mixed breed	4	Spayed female	NT	< 1.0	+	0.2	NT	suspected NRIMA (refractory)	Predonisoone, MMF, IVIG, leflunomide, Blood transfusion	Predonisoone, MMF, leflunomide	2
5	Miniature dachshund	12	Castrated male	NT	< 1.0	None	0.1	NT	suspected NRIMA (refractory)	Predonisoone, MMF, Blood transfusion	Predonisoone	2

NT, not tested.

### Study design

This study was a pilot study to assess the treatment effect of canine allogeneic ADSCs on canine refractory nonregenerative anemia. Dogs who met the inclusion criteria were treated with allogeneic ADSCs. On the day of transplantation for clinical use, the stocked cells were defrosted in DMEM (FUJIFILM Wako Pure Chemical Corporation), and MSCs were washed with saline buffer (Otsuka Pharmaceutical Factory, Inc, Naruto, Japan) three times. 1 × 10^6^ cells/kg of ADSCs were adjusted at 1 × 10^6^ cells in 5 ml of saline, and injected intravenously constant rate infusion over 1 hour within 2 h after thawed. The therapeutic effect was measured using overall clinical symptoms, laboratory tests, and need for blood transfusion.

## Results

**Fig. 1:**
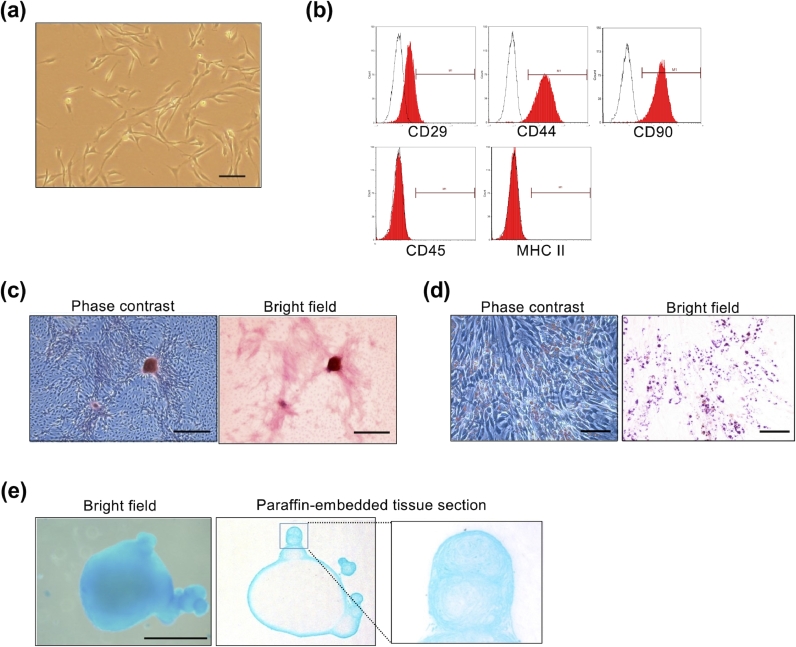
Characterization of ADSCs. **a** Light microscopic observation of ADSCs on day 7. Scale bar indicates 100 µm.; **b** Flow cytometric analysis of ADSCs. After thawed, ADSCs were positive for CD29, CD44 and CD90, and negative for CD45 and MHCII.; **c** Differentiation into osteoblasts was verified by staining with Alizarin Red S stain 14 days after change to the mesenchymal stem cell osteogenic differentiation medium. Red areas indicated the aggregates and calcinifations. Scale Bar indicates 100 µm.; **d** Differentiation into adipocytes was veriried by staining with Oil Red O stain 14 days after changing to the mesenchymal stem cell adipogenic differentiation medium 2. Lipid droplets in the cytoplasm were stained in red color. Scale bar indicates 100 µm.; **e** Differentiation into chondroblasts was verified by staining with Alcian Blue Stain 22 days after changing to the mesenchymal stem cell chondrogenic differentiation medium. Mature differentiated chondrocytes were surrounded by the cartilage substrate stained with Alucian Blue. Scale bar indicates 100 µm.

At first, allogeneic ADSCs were prepared and stocked for clinical usage. After 7 days of culture of adipose-derived cells, the cells attached to the flask, and shown to be fibroblast-like morphorogy (Fig. 1A). Cell numbers increased from 1000 cells/cm^2^ to 1.75 × 10^5^ cells/cm^2^ in 7 days culture. For characterization of ADSCs, the thawed cells from frozen stocks showed 92.9% viability. Surface antigen expression on ADSCs confirmed by flow cytometry (Fig. 1B) showed that the majority of the cells were strongly positive for MSC-positive markers, CD29, CD90 and CD44, whereas CD45 and MHC class II, negative markers for MSC, were undetectable. The multipotency of ADSCs were confirmed by the diferentions to osteoblasts (Fig. 1C), adiopocytes (Fig. 1D), and chondroblasts (Fig. 1E).

**Fig. 2:**
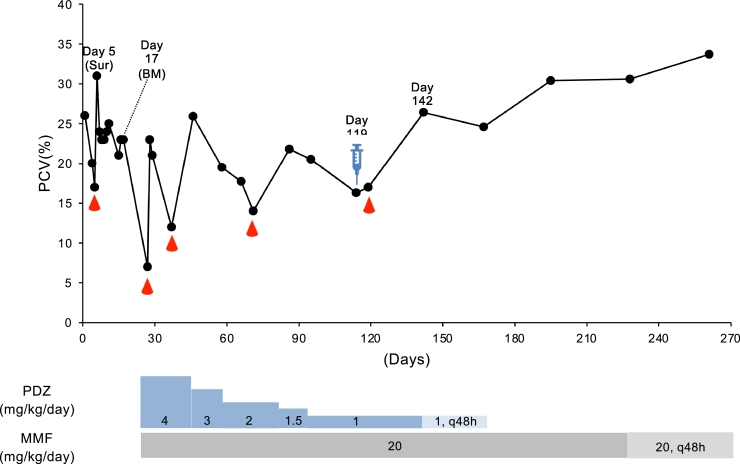
Change in PCV (%) from day 1 (first day of admission to our hospital) until last follow-up in cases 1. Droplet symbols in red color indicate the blood transfusion. Syringe symbol indicates the day of ADSC transfusion. Sur, Surgery; BM, bone marrow examination; PDZ, prednisolone; MMF, mycophenolate mofetil.

Information on the diagnosis of all cases is provided in Table 1. Case 1 was a 4.86 kg, 12 -year-old female miniature dachshund, who was evaluated for anemia that began 5 months ago and was treated with human recombinant erythropoietin at the private hospital without any diagnosis (Table 1). An initial CBC at our hospital disclosed a regenerative anemia (PCV, 26%; reference: 37%–55%), increased alanine aminotransferase (ALT) (179 U/L; reference: 17–78 U/L), alkaline phosphatase (ALP) (624 U/L; reference: <254 U/L), and C-reactive protein (CRP) (2.75 mg/dl; reference: <1.0 mg/dl). Direct Coombs’ test showed positive (× 64 at 4 °C). The spleen was found to exhibit several echogenic masses on ultrasonography, and a fine needle biopsy revealed many lymphoblastoid cells. We concluded that this dog presented with splenic lymphoma, which caused secondary immune-mediated hemolytic anemia (IMHA). After a blood transfusion on day 5, a splenectomy was performed (Fig. 2). However, the histopathological results showed the hyperplasia of white pulp and extramedullary hematopoiesis. Furthermore, anemia did not improve even after the splenectomy, which could be because regeneration of red blood cells seen before surgery was nonregenerative. On day 17, a bone marrow examination was performed to reveal the cause of nonregenerative anemia. Bone marrow (BM) examination showed slight myelofibrosis and erythroid hypoplasia. Based on this result, this dog was diagnosed with NRIMA, and then, we started the treatment with prednisolone (2 mg/kg q12h) and mycophenolate mofetil (MMF) (10 mg/kg q12h), but blood transfusion was necessary on regular basis about once a month. On day 119, we considered the continuous immunosuppressive therapy ineffective, and ADSCs were infused at 1 × 10^6^ cells/kg, followed by blood transfusion on the next day. Twenty-three days later (day 142), PCV increased to 26%, and after that, it never went down and gradually increased to 31% on day 228, despite the gradual decrease of prednisolone and MMF.

**Fig. 3:**
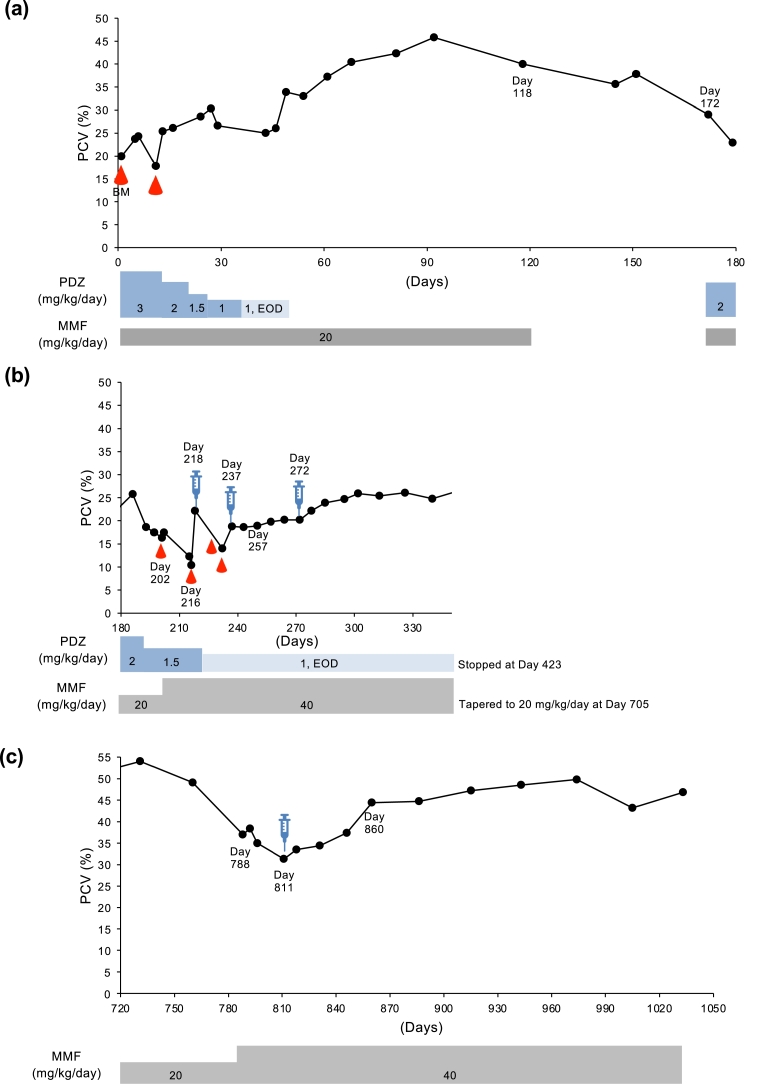
Change in PCV (%) from day 1 (first day of admission to our hospital) to day 180 (a), from day 180 to day 320 (b), and from day 720 to day 1050 (c) until last follow-up in cases 2. Droplet symbols in red color indicate the blood transfusion. Syringe symbol indicates the day of ADSCs transfusion. BM, bone marrow examination; PDZ, prednisolone; MMF, mycophenolate mofetil.

Case 2 was a 4.3 kg, 9-year-old castrated male miniature dachshund and was referred from a private hospital for examination of nonregenerative anemia that had been progressing for a month despite the treatment of immunosuppressive doses of glucocorticoid (Table 1). A blood test at his first visit to our hospital disclosed a nonregenerative anemia (PCV 20%), increased blood urine nitrogen concentration (80.1 mg/dl; reference: 9.2–29.2 mg/dl), ALT (445 U/l), and ALP (1984 U/l). On the same day as a blood transfusion, BM tests were performed. A BM examination revealed normal myelopoiesis and erythropoiesis, and this dog was diagnosed with NRIMA based on this examination and nonregenerative anemia in the peripheral blood. We began the initial immunosuppressive treatment with prednisolone (3 mg/kg PO q24h) and MMF (10 mg/kg PO q12h), and the anemia improved over the first two months, even after prednisolone was tapered (Fig. 3A). On day 118, MMF was discontinued. However, on day 172, PCV was showing a slight downward trend, we restarted prednisolone (2 mg/kg q24h) and MMF (10 mg/kg q12h) again. PCV was still gradually decreased (Fig. 3B), MMF dose was increased to 20 mg/kg q12h on day 202, and blood transfusions were performed on days 202 and 216. Due to this relapse, considering that immunosuppressive therapy did not work, we injected 1.0 × 10^6^ cells/kg of ADSCs on day 218. After 20 days, PCV was still 17%, and ADSCs was administered again (day 237) after two blood transfusions on day 225 and 233. After 3 weeks, on day 257, PCV remained 21% without going down. After one more injection of ADSCs on day 272, PCV was maintained around 25% for more than 200 days and gradually increased, resulting in 46% on day 677. Prednisolone was stopped at day 423, and MMF dose was decreased to 10 mg/kg q12h on day 705. However, on day 788, PCV dropped to 37% again, and MMF dose increased again to 20 mg/kg q12h (Fig. 3C). However, PCV gradually decreased over 30 days, and it finally dropped to 31% on day 811. ADSCs were injected again. After this, PCV start to increase again, and on day 860, PCV reached 44% and was maintained up to the present (on day 1123, PCV 51%).

**Fig. 4:**
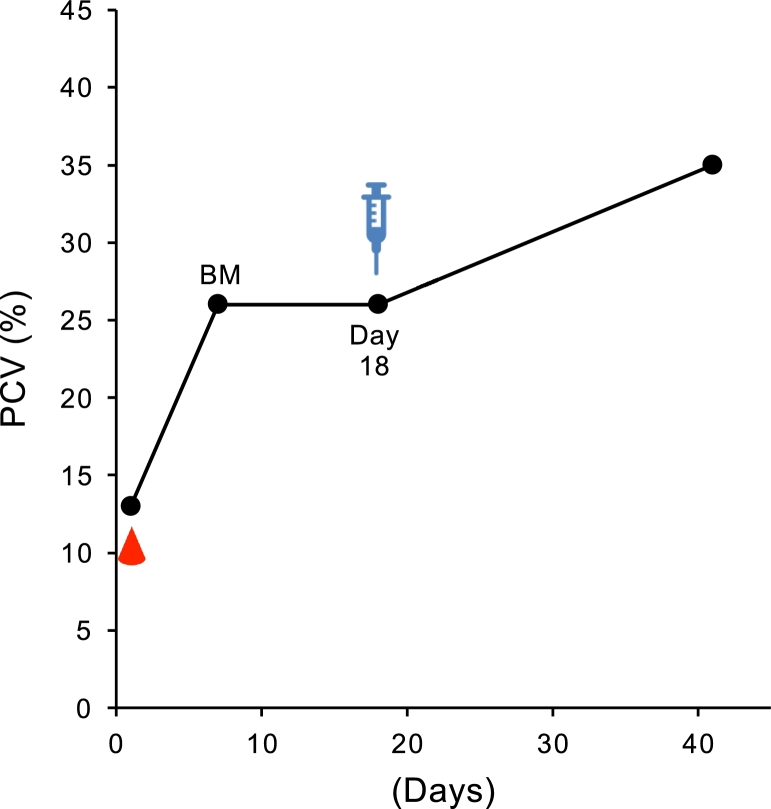
Change in PCV (%) from day 1 (first day of admission to our hospital) until last follow-up in cases 3. Droplet symbols in red color indicate the blood transfusion. Syringe symbol indicates the day of ADSCs transfusion. BM, bone marrow examination.

Case 3 was a 12.5 kg, 8-year-old female Miniature Pinscher with visible mucosal pallor and anorexia for the previous 10 days, and was referred from a private hospital (Table 1). PCV was 11% with nonregenerative anemia two days before the first visit to our hospital (Fig. 4). Prednisolone (2 mg/kg) was injected on this day and the next day in a private hospital. On the day of admission to our hospital, CBC disclosed nonregenerative anemia (PCV 13%), leukocyte sis (32,000/µl) with left shift (bands 1920/µl), monocytosis (4480/µl). The serum biochemistry profile revealed an increased ALP concentration (579 U/l) and CRP (3.30 mg/dl). Blood tests at the time of the first visit showed nonregenerative anemia and a positive Coombs’ test (× 512 positive at 37 °C and × 2048 at 4 °C). Due to the severity of the anemia, a blood transfusion was administered on that day, and a BM test was performed 5 days later to determine the cause of the nonregenerative anemia. BM examination showed erythroid hyperplasia with left shift and erythrophagocytosis and myeloid hyperplasia. Based on this result and nonregenerative anemia, this dog was diagnosed as NRIMA. At the next visit (day 18), PCV was not increased but was maintained at 26%. At the owner's request, ADSCs at 1 × 10^6^ cells/kg were administered without treatment with glucocorticoids and immunosuppressive drugs. On day 41, the PCV improved to 35%, and the patient has not visited the hospital since.

**Fig. 5:**
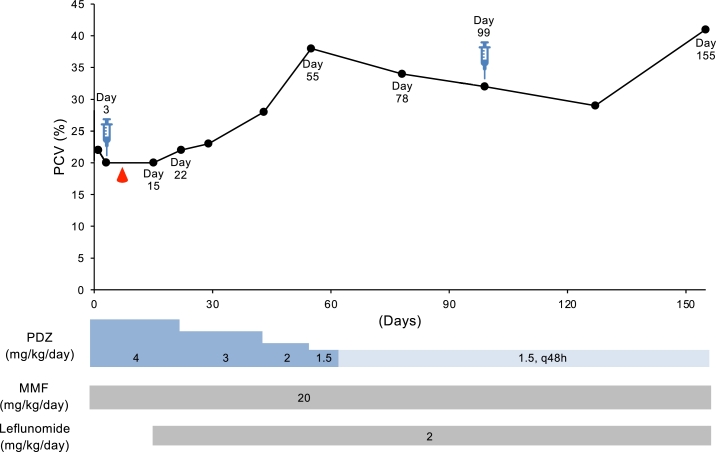
Change in PCV (%) from day 1 (first day of admission to our hospital) until last follow-up in cases 4. Droplet symbols in red color indicate the blood transfusion. Syringe symbol indicates the day of ADSCs transfusion. PDZ, prednisolone; MMF, mycophenolate mofetil.

Case 4 was a 2.76 kg, 4-year-old spayed female mixed dog with refractory anemia, and was referred from a private hospital (Table 1). Nonregenerative anemia and thrombocytopenia were discovered about a month before she arrived at our hospital, and she was tentatively diagnosed with immune-mediated thrombocytopenia and NRIMA without a BM exam. Treatment with prednisolone (2.0–4.0 mg/kg/day), intravenous immunoglobulin, and MMF (20 mg/kg q12h) improved thrombocytopenia but not anemia. Just before the first visit to our hospital, she was treated with prednisolone (4.0 mg/kg q24h), MMF (20 mg/kg q12h), and blood transfusions several times, for 2 weeks. An initial CBC disclosed anemia (PCV, 22%) with mild regeneration and leukocytosis (649.9 × 10^2^/µl) with neutrophilia (578.4 × 10^2^/µl). On this day, her general condition was good, and we continued the treatment and monitor her. However, two days later (day 3), PCV decreased to 20% (Fig. 5), and current immunosuppressive therapy was considered to be ineffective. The owner came to our hospital wishing to participate in the clinical trial, and ADSCs (1 × 10^6^ cells/kg) was infused on the tentative diagnosis of NRIMA, followed by blood transfusion on day 6. On the next visit (day 15), PCV did not increase, and leflunomide (2.0 mg/kg q24h) was added to the treatment. One week later (day 22), PCV started to increase (22%) and continued to increase, eventually reaching 38% (day 55). With the gradual reduction of the dosage of prednisolone, PCV gradually declined again over 40 days, and ADSCs were injected again on day 99. Even though the other prescriptions remained unchanged since day 78, PCV recovered to 41% on day 155. On day 255, prednisolone 1.0 mg/kg (q96h), MMF 20 mg/kg q12h, and leflunomide 2.0 mg/kg q48h were used to keep PCV at 39%.

**Fig. 6:**
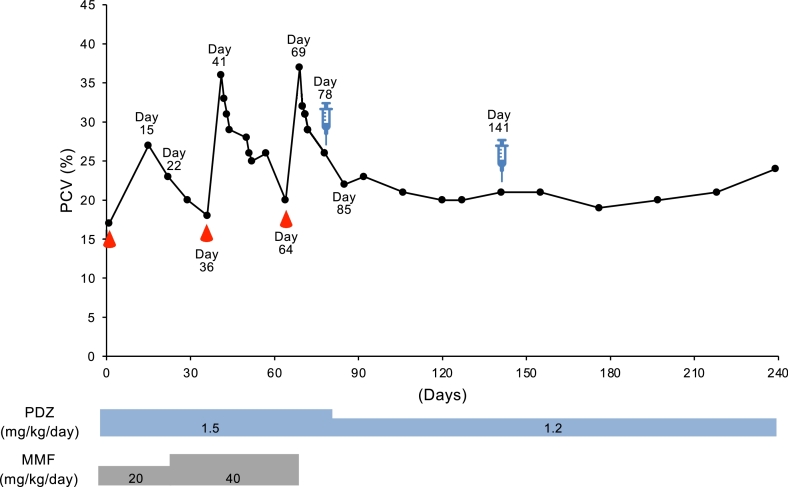
Change in PCV (%) from day 1 (first day of admission to our hospital) until last follow-up in cases 5. Droplet symbols in red color indicate the blood transfusion. Syringe symbol indicates the day of ADSCs transfusion. PDZ, prednisolone; MMF, mycophenolate mofetil.

Case 5 was a castrated male miniature dachshund weighing 9.7 kg and 12 years old, and was referred from a private hospital for treatment of refractory anemia (Table 1). Despite treatment with prednisolone 1.0 mg/kg q24h and cyclosporine 10 mg/kg q24h, the anemia progressed slowly over three months. An initial blood examination disclosed nonregenerative anemia (PCV, 17%; reticulocytes, 42,800/µl) and increased ALP (3010 U/L). On this day, blood transfusion was performed, and the immunosuppressive therapy with prednisolone (1.5 mg/kg q24h) and MMF (10 mg/kg q12h) was initiated based on the tentative diagnosis of NRIMA (Fig. 6). On day 15, PCV increased to 27% but decreased to 23% on day 22, and then, MMF was increased to 20 mg/kg q12h. On day 36, PCV decreased to 18%, and blood transfusion was performed. On day 41, PCV increased to 36%, but it gradually decreased to 20% on day 64. Then, blood transfusion was performed. On day 69, PCV increased to 37%, but as the downward trend was the same as last time, the remission with immunosuppressive therapy was considered to be difficult. Then, ADSCs at 1 × 10^6^ cells/kg were administered on day 78 (PCV 26%). At the same time, prednisolone was decreased to 1.2 mg/kg. On day 85, PCV was decreased to 22%, but after that, the PCV did not drop significantly and remained at about 20% without any blood transfusion. On day 141, ADSCs were administered again for expecting more increase in PCV. It has not risen obviously since then but has been maintained without the need for any blood transfusions.

## Discussion

In this study, we administered allogeneic ADSCs to dogs with refractory immune-mediated regenerative anemia and evaluated their response as MSC therapies for immune-mediated diseases in dogs. We used allogeneic ADSCs, instead of autologous ADSCs in order to accurately determine the outcome of the clinical trial using the same products to all dogs. The advantage of using autologous cells is that it is less immunogenic, but the advantages of allogeneic cells are that it can be prepared as a homogeneous formulation, can be used immediately by simply dissolving the stored product, and was shown to be stronger immunosuppressive effects than autologous cells at least *in vitro* (Wi et al., 2021). A clinical trial using the allogeneic ADSCs gives the less possibility of the variability of individual cells, but the possible immunogenecity. However, ADSCs were generally considered to be less immunogeneic and, in fact, there were no reports about adverse events due to the immunogenicity of allogeneic ADSCs. In this study, no adverse events were observed in any of the cases treated with ADSCs. Especially in cases 2, 4, and 5, despite multiple doses, not only were there no adverse events, but the ADSCs also appeared to be effective. This also supported that allogeneic ADSCs showed few immunogenicity as well as safety.

The current case series includes three cases where NRIMA was diagnosed by BM examination, based on the presence of nonregenerative anemia in the periphery and rubriphagocytosis or erythroid hypoplasia with inappreciable rubriphagocytosis with or without marked fibrosis on BM examination (Stokol, Blue & French, 2000; Woolhead et al., 2021). Another two cases where clinically suspected NRIMA was not confirmed by BM examination because the owner's consent has not been obtained. However, these two cases were included in this study because they were highly suspected to have NRIMA due to non-regenerative anemia in the peripheraly, history, breed, and exclusion of other diseases. Case 4, for whom a definitive diagnosis has not yet been made, originally presented with nonregenerative anemia and immune-mediated thrombocytopenia at a private hospital, so the nonregenerative anemia is also likely to be immune-mediated. Case 5 demonstrated nonregenerative anemia, and other causes of nonregenerative anemia were ruled out. The fact that the dog was a miniature dachshund, a breed predisposed to NRIMA in Japan (Tani et al., 2020), increased the likelihood of NRIMA.

NRIMA is a relatively common nonregenerative anemia in dogs and is thought to be caused by abnormal erythroid differentiation or immune-mediated destruction, but its pathogenesis remains unknown. NRIMA typically responds to immunosuppressive therapy with high-dose glucocorticoids and immunosuppressive drugs, but some cases do not respond to these drugs (Tani et al., 2020; Woolhead et al., 2021), so it is still unclear whether the disease is caused only by immune-mediated. The time to treatment response also varies, with some cases responding early and others showing an increase in PCV after several months. Therefore, determining whether the improvement in anemia caused by ADSCs administration during simultaneous treatment with immunosuppressive drugs in cases of this study is purely due to the effect of ADSCs is very difficult.

Despite immunosuppressive therapy, Case 1 required regular blood transfusions; however, after ADSCs administration, blood transfusions were no longer required, and PCV gradually increased, allowing a reduction in the amounts of immunosuppressive drugs, indicating that ADSCs was effective. Case 2 appears to have responded to two doses (days 218 and day 237) of ADSCs, but we cannot deny the possibility that the PCV was maintained due to the increased dose of MMF shortly before that. However, the increase in PCV after the third dose of day 272 was considered to be the effect of ADSCs alone, since nothing else was changed. Determining whether the subsequent increase in PCV was the effect of ADSCs administration alone is difficult since the MMF dose was increased one month before the fourth dose on day 811. Case 3 was the only case in this study in which MSCs were treated solely with MSCs. However, because the PCV was 26% on the day of the BM examination performed after the transfusion and no decrease was found in PCV 10 days later, we cannot rule out the possibility that the patient was already on an improving trend for some reason. Conversely, NRIMA does not cause rapid destruction of red blood cells, so it may have just been maintained by transfusion. In case 4, ADSCs were administered on day 3, and after ADSCs administration, PCV did not increase immediately, so leflunomide was added, and PCV increased from one week later. Therefore, it cannot be denied that the addition of leflunomide may exhibit an effect. However, from day 55 to day 99, mild anemia considered to be a recurrence was found, with PCV gradually increasing to 40% after ADSCs monotherapy without any changes in other treatments, indicating that the patient responded to ADSCs. In case 5, the patient did not respond to immunosuppressive treatment and required regular blood transfusions; however, the ADSCs were partially effective because no blood transfusions were required following the administration of the ADSCs, although the PCV did not increase significantly. It took a certain amount of time from the administration of ADSCs to recover in cases of this study. This is as a result that it takes longer for NRIMA to respond to treatment than IMHA (Stokol et al., 2000), and it is also an indication that the therapeutic effect of ADSCs takes longer to manifest.

As ADSCs are approved and used for GVHD in humans (Murata & Teshima, 2021), the immunosuppressive or immunomodulatory effects of ADSCs are very promising. The immunosuppressive effects of ADSCs have also been demonstrated *in vitro* culture in dogs (Chow, Johnson, Coy, Regan & Dow, 2017; Teshima, Yuchi, Suzuki, Matsumoto & Koyama, 2021). Chow et al., showed ADSCs utilized TGF-β signaling pathways to suppress canine T cell activation *in vitro*. Teshima et al., found that canine ADSCs produced extracellular vesicles, which inhibited the proliferation of Th1 and Th17 and enhanced Th2 and Treg cell proliferation. Although the therapeutic effects of ADSCs reported in dogs in immune-mediated diseases such as IBD (Pérez-Merino, Usón-Casaús, Hermida-Prieto & Mariñas-Pardo, 2016) and CAD (Kaur et al., 2021; Villatoro et al., 2018), no reports exist on the therapeutic effects of ADSCs for hematological diseases, and this is the first report of its kind.

Except case 3, all dogs were received the immunosuppressive therapy when ADSCs was administered. Addition of ADSCs to the immunosuppressive drugs were reported to enhance immunosuppressive effects in transpalnts of animal models (Casiraghi, Perico & Remuzzi, 2013; Ge et al., 2009). Concurrent treatments of immunosuppressive drugs and ADSCs in this study might have some synergistic effects on immunosuppressive function of ADSCs in cases of this study. This kinds of concept needs to be further examained in double-armed clinical study in the future, since each immunosuppressant has been reported to have different effects on MSCs *in vitro* (Javorkova et al., 2018).

As mentioned earlier, some of the patients in this study have not been definitively diagnosed, and the concurrent treatments and the timing of the indication of ADSCs were not consistent. From these reasons, actual effects of ADSCs for the treatment of NRIMA were still remained. Nevertheless, as discussed earlier, if we judge the course of each case carefully, we can say that the effect of ADSC has improved chronic refractory anemia and obviously reduced the need for blood transfusions. The classification and pathogenesis of nonregenerative anemia in dogs are still unclear, but with the exception of myeloproliferative disorders and dysdifferentiation, the majority are thought to be immune-mediated destruction. As a result, prospective clinical trials are required to investigate the true therapeutic effect of ADSCs on NRIMA. The immunomodulatory effects of ADSCs are very promising, and despite numerous clinical trials in humans, many unanswered questions still exist. Therefore, the expectation exists that ADSCs will be used in dogs to treat various immune-mediated diseases, in order to investigate their effects.

## Conclusions

Three cases with NRIMA or two cases suspected with NRIMA were treated with allogeneic ADSCs. Although immunosuppressants were concurrently taken except one dog, allogeneic ADSCs improved refractory anemia and another blood transfusion was nevere needed. This study indicates the possibility of allogeneic ADSCs as a rescue treatmenet for treatment of canine immune-mediated anemia.

## Authors’ contributions

All authors examined cases. T.M. supervised work interpreted data and wrote the manuscript. All authors read and approved the manuscript.

## Data availability statement

The datasets in this study are available from the corresponding author on reasonable request.

## Funding

T.M. received research funding from FUJIFILM Corporation.

## Ethics approval and consent to participate

All examinations were performed with informed owner's consent according to ethical guidelines of the Yamaguchi University Animal Medical Center.

## Consent for publication

Informed written consent to publish all given information was obtained of all five owners of the presented dogs.

## Clinical trial registration

No.5 of the Ethics Review Board of the Joint Faculty of Veterinary Medicine of Yamaguchi University. Registered 20 Feburary 2018.

## Declaration of Competing Interest

T.M. received research funding from FUJIFILM Corporation. KK was associated with Anicom Specialty Medical Institute Inc., Japan*.* The remaining authors declare that the research was conducted in the absence of any commercial or financial relationships that could be construed as a potential conflict of interest.
